# Mother’s Knowledge, Attitude, and Practice Toward the Prevention and Home-Based Management of Diarrheal Disease Among Under-Five Children in Kurdistan Region of Iraq

**DOI:** 10.7759/cureus.76186

**Published:** 2024-12-22

**Authors:** Amir K Saleh, Farhad Armishty, Piran Hossam, Hashem Melhim, Qusay Nawaf

**Affiliations:** 1 Medicine, University of Zakho, Zakho, IRQ; 2 Clinical Sciences, University of Zakho, Zakho, IRQ

**Keywords:** attitude, diarrhea, knowledge, mothers, practice

## Abstract

Background

Diarrhea is defined as three or more loose or watery bowel movements per day and any additional bowel motions that mothers deem abnormal or extra frequent in children. It is important to note that among children in underdeveloped countries, diarrhea is one of the main causes of illness and death. Severe diarrhea causes significant fluid loss and can be fatal. The primary goal of this study was to evaluate the mothers' knowledge, attitude, and behavior about the home-based care of diarrhea in Zakho, Duhok, Kurdistan, Iraq, for children under the age of five.

Methodology

The study was cross-sectional in design. The study period was extended from March 1 to April 1, 2022. The study was conducted in Zakho cities in Kurdistan, which is located in the north of Iraq. The data were evaluated using a statistical tool for social sciences (SPSS, version 26; IBM Corp., Armonk, NY) and two different methods. We conducted our research through in-person interviews.

Results

A total of 400 mothers participated in the survey, and 100% responded. Thus, the analytical method contained the data from 400 respondents. In this study, we found that only 233 (58.25%) of the mothers had good practice in the prevention and home-based treatment of diarrheal diseases in children under the age of five, whereas 282 (70.5%) of the mothers had good knowledge and 208 (52%) had a positive attitude.

Conclusion

This research showed that 70.5% of the mothers had excellent knowledge about the prevention and home-based treatment of diarrheal diseases. In terms of attitude, 52% showed a good attitude toward the prevention and home-based management of diarrhea, and 58.25% of the mothers who participated in this study had good practices for prevention and home-based care of under-five diarrhea.

## Introduction

Diarrhea is defined by the World Health Organization (WHO) as having three or more loose or watery bowel movements per day, as well as any additional bowel motions that mothers deem abnormal or extra frequent in children [1-3]. Clinically, diarrhea can be categorized as acute bloody diarrhea, acute watery diarrhea, or chronic diarrhea, lasting at least 14 days [4,5].

It is worth mentioning that one of the main factors contributing to morbidity and death among children in developing nations is diarrhea [5-8]. According to mortality estimates in underdeveloped nations, diarrheal illnesses claim the lives of 4.9 children per 1,000 per year in the first five years of life [9]. With the majority of deaths happening in the first year of life, under-five-year-old children's mortality is accounted for by this in 21% of cases [9]. Iraq has a young population, with 45% of people under the age of 15 and 17% of the population (3.9 million) under the age of five. Two-thirds of people reside in cities [4]. As a result, diarrhea is the second most common reason for child deaths in Iraq [5,10].

Diarrhea is also a key contributor to malnutrition, which puts them at risk for other infectious diseases that raise child mortality rates in these nations [9]. Severe diarrhea causes significant fluid loss and can be fatal, particularly in young infants who are underweight or have compromised immune systems [9].

Infected diarrhea is a key factor in child mortality in impoverished countries where access to clean water and waste disposal is frequently poor or nonexistent [11]. Iraq's dysfunctional healthcare system and ongoing public health crises are having an impact on the most vulnerable populations, including children. This is due to the effects of the war, sanctions, and sectarian violence [10].

The most frequent causes of acute diarrhea in children include several viral, bacterial, and parasitic illnesses [10]. About 70% of cases of contagious diarrhea in children are brought on by viruses [9]. The most frequent one is rotavirus; incidence rates are comparable in industrialized and developing countries. Admitted children in hospitals in Basra, Erbil, Tikrit, and Baghdad experienced acute diarrhea at a rate of 24%, 37%, 18.5%, and 30%, respectively, due to rotavirus [9].

Access to clean water, better sanitation practices, hand washing with soap, breastfeeding exclusively for the first six months of life, good personal and food hygiene, awareness of infection transmission, and rotavirus vaccination are some crucial preventative measures for diarrhea that are required for both prevention and treatment [2]. Handwashing with soap is one of the most affordable public health treatments that can potentially reduce diarrhea by 23% to 48% [12].

The UNICEF and WHO organizations established Oral Rehydration Salts (ORS) and Oral Rehydration Treatment (ORT) in the late 1970s, and both have proven effective in treating pediatric diarrhea. According to estimates, the marketing and usage of these treatments may have avoided more than one million diarrhea-related deaths annually in the 1990s. However, there are signs that understanding and usage of effective at-home treatments for diarrhea, including ORT, may be waning in some countries today [13,14].

Although diarrhea may not always result in death, mothers' wrong beliefs, bad habits, and misguided methods for managing and preventing it lead to high levels of severe dehydration and, eventually, fatality [1]. Therefore, the primary goal of this study was to evaluate the mothers' knowledge, attitude, and behavior about the home-based care of diarrhea in Zakho, Duhok, Kurdistan, Iraq, for children under the age of five.

## Materials and methods

Study area and period

A cross-sectional study was conducted in Zakho General Hospital in the Iraqi city of Zakho, Kurdistan, from March 1, 2022 to April 1, 2022. The mothers of 400 children who took part in this study ranged in age from 15 to 45. 

Study design and participants

A study was conducted in Zakho general hospitals to evaluate mothers' knowledge, attitudes, and behavior regarding the prevention and home-based care of diarrheal illness among children.

Measurement and data collection procedure

Face-to-face interviews were employed, using a standard and structured questionnaire that contained socio-demographic status, knowledge, attitude, practice, and health-seeking behavior questions of the mothers regarding under-five children with diarrheal diseases. Four trained college students who collected data were present.

Data processing and analysis

The data collectors, investigators, and supervisor carefully examined each questionnaire once data collection was finished to ensure its accuracy and consistency. The data was analyzed with SPSS for Windows version 26 (IBM Corp., Armonk, NY). The frequency, percentages, and mean of the study's findings were determined using descriptive statistics. The findings were presented using tables, graphs, and outcome statements.

Inclusion Criteria

Mothers who have children younger than five years have previous episodes of diarrhea, and mothers whose children have recent episodes of diarrhea.

Exclusion Criteria

Mothers whose children are more than five years old, infertile women, mothers who do not have children yet, and those mothers with physical impairment (unable to hear and speak) and mental illness were excluded from the study.

Ethical approval

The study proposal was approved by the ethics committee of the College of Medicine/University of Zakho in the Kurdistan Region of Iraq. Before collecting samples, parents of children were approached for permission to participate in the study, and all participants signed an informed written agreement. This approval is documented by a letter issued with the reference number (JAN29/E07) on January 12, 2022.

## Results

The study included 400 mothers in all, with a response rate of 100%. Thus, the analytical process contained the data from 400 respondents.

Social and economic status of the mothers

This study (50.7%) included mothers, who ranged in age from 25 to 34 and had a mean age of 27, who were around half that age. Muslims (69.5%) and Yazidis (29.5%) were the most common religious groups. Kurds made up 387 (96.8%) of the mothers, while Arabs made up 13 (3.3%). Of the participants, 336 (84%) were housewives, while 49 (12.3%) were employed. Of the children, 196 (49.5%) were between the ages of six and 24 months (Table 1). 

**Table 1:** Sociodemographic details of the respondents in Kurdistan, Iraq, in 2022

Characteristic	Category	Frequency	Percentages
The mother's age	19-24 years	88	22
	25-34 years	203	50.7
	35-44 years	104	26
	45 years	5	1.3
The child's age	Less than 6 months	73	18.3
	6- 24 months	196	49
	24-60 months	131	32.8
The mother's occupation	Housewife	336	84
	Employed	49	12.3
	Self-employed	15	3.8
Level of education of the mother	Unable to write and read	161	40.3
	Primary school	99	24.8
	Secondary school	80	20
	Diploma	60	15
The mother’s religion	Muslim	278	69.5
	Christian	4	1
	Yazidi	118	29.5
The mother’s ethnicity	Kurd	387	96.8
	Arab	13	3.3

Knowledge of mothers on the prevention and treatment of diarrhea in young children

In contrast to the vast majority of mothers (93.3%) who described diarrhea as passing loose feces three or more times per day, just one (0.3%) mother saw bloody stool. Consuming tainted water is considered to be the cause of diarrhea by 322 (75.5%) of respondents. Around 24.3% of the participants selected lethargy or weakness as a dangerous sign of diarrhea in children under five. On the other hand, only five (1.3%) of them knew that a continuous demand for water is a sign of diarrheal illness (Table 2). 

**Table 2:** Maternal awareness of the top five diarrheal illnesses in Kurdistan, Iraq, in 2022

Characteristic	Category	Frequency	Percentages
Diarrhea definition	Frequent passage of watery stool (3 times or more)	373	93.3
	Normal stool passing regularly	17	4.3
	Bloody stools	1	0.3
	Greenish stools	3	0.8
	No idea	6	1.5
Diarrheal causes	Teething	83	20.8
	Evil eye	5	1.3
	Contaminated water	302	75.5
	No idea	10	2.5
Diarrheal danger signs	Becoming weak or lethargic	97	24.3
	Vomiting everything / Repeated vomiting	172	43
	Bloody stool and fever	126	31.5
	Marked thirst for water	5	1.3

During their child's diarrheal sickness, less than half of the participants (123 (30.8%)) used homemade solutions. The solution was created by each of them by combining one teaspoon of salt and eight teaspoons of sugar in one liter of water. Around (225 (56.3%)) of the mothers were aware of the appropriate water volume for mixing an ORS sachet (i.e., 1,000 mL of water to one sachet of ORS). According to Table 3, 120 of the loose respondents (30%) thought that ORS should be delivered after each stool the child passed, while 54 (13.5%) felt that it should be given anytime a child needed to drink. 

**Table 3:** Respondents' understanding of proper ORS use in Kurdistan, Iraq, in 2022

Variable	Category	Frequency	Percentages
How is ORS prepared?	1 sachet of ORS- 300 ml of water	11	2.8
	1 sachet of ORS- 500 ml of water	96	24
	1 sachet of ORS- 600 ml of water	26	6.5
	1 sachet of ORS- 1000 ml of water	225	56.3
	1 sachet of ORS- 1500 ml of water	21	5.3
	Other	21	5.3
How often should ORS be given?	After every watery stool	120	30
	Once a day	52	13
	2–3 times a day	174	43
	Whatever child wants to drink	54	13.5
How long should the mixed ORS last?	24 h. (1 day)	283	70.8
	48 h. (2 days)	66	16.5
	72 h. (3 days)	46	11.5
	96 h. (4 days)	4	1
	Other	1	0.3

Mothers' perspectives on preventing and treating diarrhea in children under five at home

**Figure 1:**
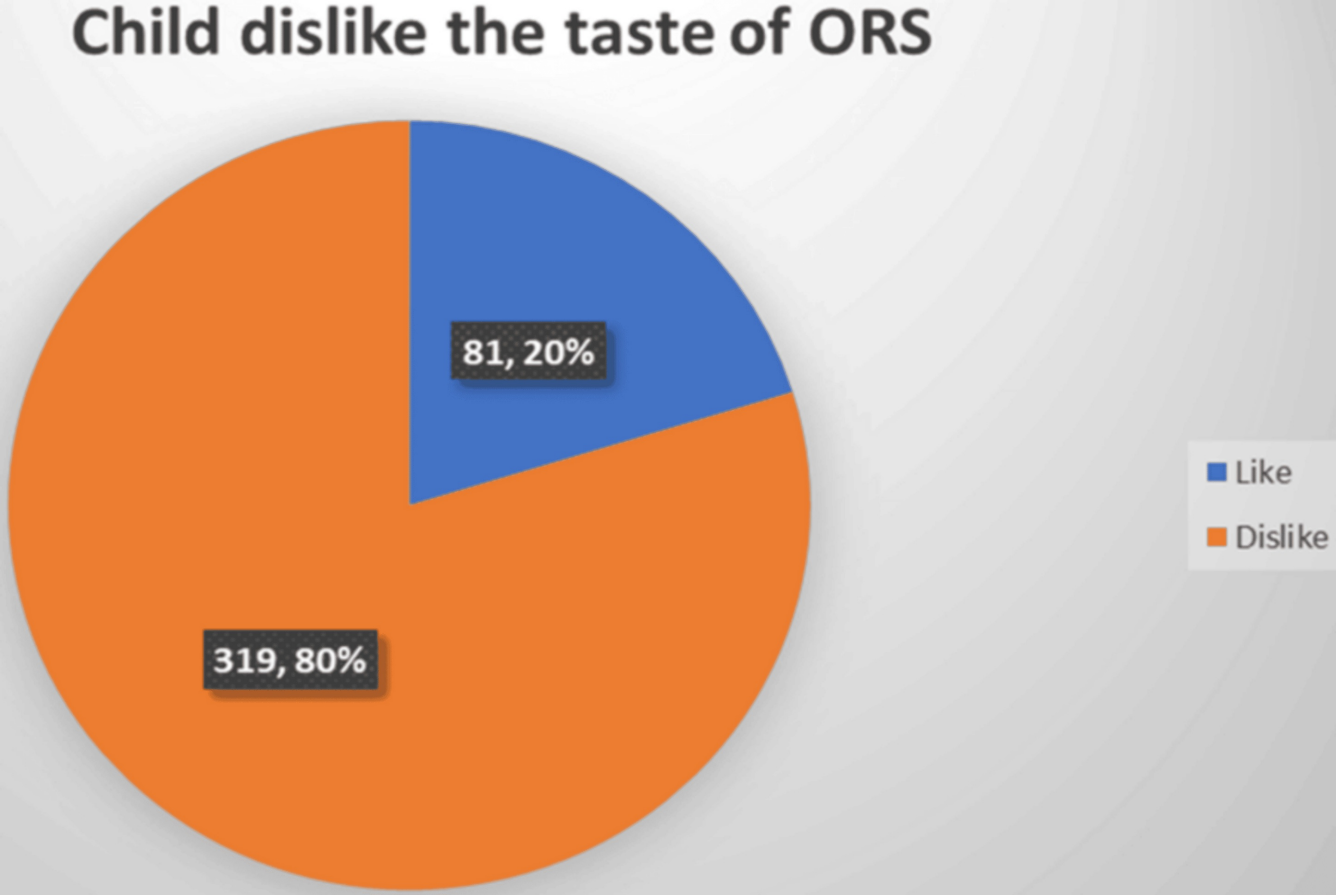
Mothers attitude about the taste of oral rehydration fluid by their children in Kurdistan, Iraq, in 2022

The majority of the respondents (226 (56.5%)) agreed that oral rehydration solution should be available at home for the treatment of children under the age of five who have diarrheal diseases. In a similar vein, 229 (57.3%) of the participants believed that “mothers can cure their children's diarrheal disease at home.” A total of 319 individuals (79.8%) reported that their children disliked the taste of the oral rehydration solution (Table 4, Figure 1).

**Table 4:** Mothers' response regarding ORS and home treatment of diarrhea

Mothers’ response	Agree	Disagree
Giving ORS at home can treat diarrhea	226 (56.5%)	174 (44%)
Mothers can cure their children's diarrheal disease at home	229 (57.3%)	171 (43%)

Mothers' methods for preventing and treating diarrhea in children under five at home

Only 199 women (29.8%) nursed their infants more frequently during the diarrheal episodes, whereas 165 mothers (41.3%) breastfed their infants less frequently. In a similar vein, most mothers (167, 42.8%) gave their children more liquids than normal while they were experiencing diarrhea, while 177, 44.3%, gave their children fewer liquids than usual. In terms of feeding, 331 (82.8%) mothers provided less food than usual, whereas 12 (3%) mothers provided more food than usual to consume during diarrheal episodes. As seen in Table 5, the majority of mothers reported washing their hands before preparing food (51.5%), after preparing food (4.8%), and after defecating (41.8%).

**Table 5:** Mothers in Kurdistan, Iraq, feed their children during episodes of diarrhea and how they wash their hands

Characteristic	Category	Frequency	Percentages
When (Name) had diarrhea, did you breastfeed him/her less than usual, about the same amount, or more than usual?	Less	165	41.3
	Same	36	9
	More	119	29.8
	Child not breastfed	58	14.5
	Don’t know	22	5.5
When (Name) had diarrhea, was he/she offered less than usual to drink, about the same amount, or more than usual to drink?	Less	177	44.3
	Same	47	11.8
	More	167	41.8
	Child not breastfed	3	0.8
	Don’t know	6	1.5
Was (name) offered less than usual to eat, about the same amount, or more than usual to eat?	Less	331	82.8
	Same	42	10.5
	More	12	3
	Child not breastfed	2	0.5
	Don’t know	13	3.3
When do you wash your hands with soap	Before food preparation	206	51.5
	Before feeding children	19	4.8
	After defecation	167	41.8
	Never	8	2

During their children's diarrheal episode, mothers' care-seeking behavior and locations During the period of diarrheal diseases, almost all of the mothers (348 (87%)) seek medical care for their children. The majority of those who needed treatment for their child's diarrhea went to a health center (50.7%), while 155 (38.8%) visited the hospital (Table 6). 

**Table 6:** Care-seeking patterns and locations of mothers in Kurdistan, Iraq, in 2022

Characteristic	Category	Frequency	Percentages
Did you seek advice or treatment from someone outside of the home for (Name’s) diarrhea?	Yes	348	87
	No	52	13
Where did you initially turn to for guidance or care?	Hospital	158	39.5
	Health center	205	51.2
	Health post	9	2.3
	Clinic	13	3.3
	Pharmacy	5	1.3
	Friends/relatives	10	2.5

Total level of mothers' knowledge, attitude, and conduct toward the treatment and prevention of diarrhea at home in children under five

By asking the mother 11 questions, we were able to gauge her degree of understanding. Suppose they were aware of ORS and its advantages. Mothers who properly answered questions above the mean were deemed to have “excellent knowledge,” whereas those who correctly answered questions below the mean were deemed to have “bad knowledge.”

Additionally, the attitude was evaluated in terms of whether they agreed or disagreed in seven questions that the primary line of therapy for diarrhea is ORS, how ORS tastes to their child, and other factors. Mothers with replies above the mean were referred to as having a “positive attitude,” while those with responses below the mean were referred to as having a “negative attitude.”

Mothers' overall conduct was evaluated in the same way as others by asking nine questions concerning how ORS is prepared, how frequently it is provided, how long a mixed ORS should be continued, and so on. Mothers were categorized as having “excellent practices” if their responses were above the mean, and those who did not were categorized as having “bad practices.”

According to these criteria, 282 (70.5%) of the mothers had excellent knowledge, whereas 118 (29.5%) had low knowledge about the prevention and home-based treatment of diarrheal diseases in children under the age of five. In terms of attitude, only 208 women (52%) showed a good attitude toward the prevention and home-based management of diarrhea in children under the age of five, compared to more than half of the mothers (192 (48%)). Only 233 (58.25%) of the mothers who participated in this study had good practices, while the remainder 167 (41.75%) had poor practices for prevention and home-based care of under-five diarrheas.

## Discussion

The purpose of this study was to assess mothers' knowledge, attitudes, and behaviors regarding the prevention and home treatment of diarrheal infections in children under the age of five in Zakho, Duhok, Kurdistan, Iraq. According to the findings, the majority of respondents (70.5% and 48%) had high knowledge and poor practice addressing the prevention and home management of diarrheal illnesses in children under the age of five.

According to the findings of this survey, 70.5% of mothers were knowledgeable about the prevention and home-based management of diarrhea in children under the age of five. This conclusion is greater than those of research conducted in Kashan, Iran, where 28.8% of mothers had a strong awareness of diarrhea and 24.7% had little understanding of diarrhea [15]. The knowledge of the mothers was related to the mother's age, the father's education, the number of children, the mother's employment, and the source of the information [15].

The majority of the mothers (93.3%) accurately characterized diarrhea (as passing loose stool three or more times per day), and a similar finding was reported in Diredawa, Eastern Ethiopia (92.2%) [1]. The findings of this study were substantially greater than those of earlier Saudi studies, with almost 49% of the mothers reporting the passing of three or more loose stools with blood during the day [16].

In terms of the causes and transmission of diarrhea, roughly 75.5% of participating mothers stated that the most prevalent cause of pediatric diarrhea was eating contaminated food and drinking contaminated water. Much research has found that mothers have a limited awareness of the causes and transmission of diarrhea, which is consistent with our findings. According to Indian research, the most prevalent cause of children’s diarrhea (28% of the time) is contaminated food [17]. Another Iranian study found that just 24.66% of women were aware that contaminated water might induce diarrhea [15]. According to a Saudi survey, 31% of women believe that contaminated water is the primary cause of diarrhea [16]. The variation in mothers' education levels may be the cause of the shift in understanding childhood diarrhea. There is a widespread perception that diarrhea and teething are related. According to the findings of the current study, 20.8% of mothers blamed their child's diarrhea on teething. These findings concur with those of previous research conducted in other nations.

The only limitations we encountered during data collection were the large number of questions and some mothers' refusal to take part in the study. According to a study from Nepal, 20.8% of mothers deemed red-colored diarrhea to be “the most dangerous diarrhea,” and our study reveals that approximately 172 (43%) of the mothers thought that repeated vomiting or vomiting everything was a dangerous sign associated with diarrhea. Additionally, approximately 126 (31.5%) of the mothers thought that it might be associated with fever and blood in the stool [18].

The main treatment for diarrhea, according to the Integrated Management of Childhood Illness (IMCI) recommendations, is the administration of ORS [19] Mothers' usage of ORS also appeared to be quite positive. Over 90% of the mothers who took part in our study were aware of the ORS, and more than half of them - 82.2% - used it for their children's sake. Since 62% of the participating mothers in Saudi Arabian research knew about the ORS, our study may reflect greater findings from that study [16]. Only 23.5% of them utilized it for children, while another Pakistani survey found that while 58% of women were aware of ORS, only 27% of them used it on children [20].

The majority of mothers also concurred that ORT is the primary treatment of choice for diarrhea and that it may restore lost liquids. The majority of the mothers in the Saudi research agreed that ORT can replenish lost fluids, but they disagreed that ORT should be the first-choice treatment for diarrhea [16].

In this study, 44.3% and 82.8% of the mothers fed their children less often and supplied fluids to them during diarrheal illnesses. Similar to this, more than 61.4% and 62.7% of mothers in Diredawa, Eastern Ethiopia, offered fluid and feeding to their child less frequently than usual during the diarrheal episodes [1], as did 70% of mothers in Kenya and 19.6% of mothers in India [21,22]. The majority of the mothers in this research region lacked formal education, which may be the main cause of the gap as these mothers were unable to access books, newspapers, and other reading materials. The mothers' concern about experiencing further vomiting and losing more watery stools is another reason why they may have reduced their fluid intake and feedings when their children were experiencing diarrhea.

The secret to managing childhood diarrhea at home is for mothers to have sufficient information on the causes, prevention, and management of diarrhea using appropriate remedies. The majority of the participating mothers (87%) seek outside guidance or care for their children who are experiencing diarrhea, with roughly 35.6% of them visiting a hospital and about 54.5% going to a health center. Similar results were seen in South Arabia, where 68.9% of participating mothers sought medical attention for their children who had diarrhea [16].

The majority of mothers (51.5%) often wash their hands before making food and (41.8%) after defecating. In Diredawa, Eastern Ethiopia, it was discovered that most mothers (67.8% and 100%) often wash their hands before making meals and after defecating, respectively [1]. In Bangladesh, 60.0 and 3.1% of people, respectively, do not wash their hands before preparing meals or after defecating [23]. It is possible that this variety results from cultural, sociodemographic, and information access disparities.

Study limitation

The only limitations we encountered during data collection were the large number of questions and some mothers' refusal to take part in the study.

## Conclusions

This research showed that 70.5% of the mothers had excellent knowledge about the prevention and home-based treatment of diarrheal diseases. In terms of attitude, 52% showed a good attitude toward the prevention and home-based management of diarrhea, and 58.25% of the mothers who participated in this study had good practices for prevention and home-based care of under-five diarrhea.

More research is required to educate mothers of children under five on how to treat diarrhea at home in order to lower the morbidity and mortality rate among this age group. Health education should be provided to mothers on how to make ORS, make fluids at home, avoid diarrhea, and identify signs of dehydration. Educating the public about the value of nutrition during diarrheal outbreaks by launching awareness campaigns and disseminating health information through the media. Children's development, intellectual growth, and physical growth all depend on health information.
